# Design and Clinical Evaluation of a Non-Contact Heart Rate Variability Measuring Device

**DOI:** 10.3390/s17112637

**Published:** 2017-11-16

**Authors:** Jure Kranjec, Samo Beguš, Gregor Geršak, Matjaž Šinkovec, Janko Drnovšek, Domen Hudoklin

**Affiliations:** 1 Faculty of Electrical Engineering, University of Ljubljana, Tržaška 25, 1000 Ljubljana, Slovenia; jure.kranjec@fe.uni-lj.si (J.K.); samo.begus@fe.uni-lj.si (S.B.); gregor.gersak@fe.uni-lj.si (G.G.); janko.drnovsek@fe.uni-lj.si (J.D.); 2 Clinical Department of Cardiology, University Medical Centre Ljubljana, Zaloška 7, 1000 Ljubljana, Slovenia; matjaz.sinkovec@kclj.si

**Keywords:** heart rate measurement, heart rate variability, non-contact, ultrasound, clinical evaluation, target uncertainty

## Abstract

The object of the proposed paper is to design and analyze the performance of a non-contact heart rate variability (*HRV*) measuring device based on ultrasound transducers. The rationale behind non-contact *HRV* measurement is the goal of obtaining a means of long term monitoring of a patient’s heart performance. Due to its complexity as a non-contact measuring device, influential physical quantities, error source and other perturbations were thoroughly investigated. For medical purposes it is of utmost importance to define the target uncertainty of a measuring method from the side of physicians, while it is the role of scientists to realistically evaluate all uncertainty contributions. Within this paper we present a novelty method of non-contact *HRV* measurement based on ultrasound transducers operating at two frequencies simultaneously. We report laboratory results and clinical evaluations are given for healthy subjects as well as patients with known heart conditions. Furthermore, laboratory tests were conducted on subjects during a relaxation period, and after 1 min physical activity

## 1. Introduction

The heart rate (*HR*) and its derivate, the heart rate variability (*HRV*), have been recognized as one of the most important markers in the relationship between the autonomic nervous system (ANS) and cardiovascular mortality [1]. Further, the analysis of these nonstationary and nonlinear parameters provide a reliable reflection of many physiological factors modulating heart rhythm (e.g., binary symbolization of RR interval dynamics gives a better understanding of normal heart period regulation, the amount of *HR* fluctuations around the mean *HR* can be used as a mirror of the cardiorespiratory control system, time dependent spectral *HRV* analysis using the wavelet transformation was found to be valuable for explaining the patterns of cardiac rate control during reperfusion, etc.). As such they are a powerful means for observing the interplay between the sympathetic and parasympathetic nervous systems [2], indicating impending cardiac diseases, which may lead to a sudden cardiac arrest [1].

The *HRV* has so far been put to use in different clinical applications, such as diabetes, arterial hypertension, diabetic neuropathy, etc. [1,2,3]. The two most common non-invasive methods used for measuring heart activity are the electrocardiogram (ECG) and plethysmography (PPG). The ECG device is used to record the electrical activity of the heart via electrodes in contact with patient’s skin. By altering the shape of its constituent waves (P, QRS and T) the method conveys information about the heart’s activity and possible conditions in a standardized format [4]. The PPG on the other hand is an optical measurement, detecting changes in blood flow reflected by a pulsed wave. The PPG heart rate measurement is obtained by means of a pulse oximeter attached to the tip of a finger or to an earlobe, illuminating the skin and measuring changes in light absorption of the peripheral tissue [5,6]. While both methods are proven and reliable, both also have drawbacks, such as susceptibility to motion artefact, need for physical contact, possibility of allergic reactions to gels, limitation of subject’s mobility due to electrode sensors, may not be appropriate for specific groups of patients (burn injuries, infants), etc. [6,7,8,9]. Furthermore, obtaining data from an ECG depends partially on electrode placement and site preparation so it can reduce impedance between the electrode and the skin surface. High impedance can be caused by several factors, such as dry skin, presence of hair, calluses, scar tissue etc. [10].

Both *HR* and *HRV* are amongst the most commonly used parameters in health care. Due to their importance and their relatively easy derivation from non-invasive and commercially available equipment, other fields and non-medical applications are showing high interest of parameter monitoring in real life conditions. Some such recent examples include studies of the psychophysiological burden of athletes [11,12,13,14], air traffic controllers [15], drivers [16,17], rehabilitation, Posttraumatic Stress Disorder patients [18,19,20], etc.

Due to the mentioned drawbacks within the existing measurement processes and ever increasing number of possible medical, research and commercial fields of applications, researchers worldwide are exploring the possibilities of experimental non-contact measuring techniques. Several promising novel methods are emerging with encouraging results [21,22,23,24].

In the past, a review study of non-contact experimental methods was presented [7], where several of most promising non-contact methods were taken into account. In addition, a feasibility study of some methods was performed, indicating that non-contact measurement of *HR* and its derivate *HRV* is possible, especially for distances of less than 50 cm [21]. The goal of this study is to perform and present a feasibility study with proof of concept of an assembled non-contact US measuring device. *HR* will be measured an ultrasound generated signal, on a group of healthy volunteers in a laboratory environment and lastly on volunteers with known cardiac pathology in a clinical environment under surveillance of medical staff. For the latter two cases, time domain *HRV* parameters are also given. The test measurements on subjects were performed in a state of relaxation and after physical activity in laboratory conditions. The measurement of the device was compared to a reference ECG measuring device (MP150 acquisition & analysis system with III-lead ECG100C ECG amplifier by BIOPAC Systems, Inc., Goleta, CA, USA).

## 2. Methodology

### 2.1. III-Lead ECG As a Reference HR(V) Measuring System

As the reference *HR* measurement method, a III-lead ECG system was used. The reference system used in our experiments was the Biopac MP150 acquisition & analysis system. It is a flexible 16-channel device, able to record multiple channels with different sample rates at speeds up to 400 kHz (aggregate). Due to its modularity and powerful automated analysis (available for ECG, *HRV*, EEG, EMG, EGG, etc.) it is applicable to over 40 research fields and is already cited in over 27,000 journal articles & scholarly references [25]. In combination with the MP150 main unit, the ECG100C unit was used. The ECG100C unit is a single channel, high gain, differential input, biopotential amplifier designed specifically for monitoring the heart’s activity [26,27].

### 2.2. Ultrasound Sensor As Experimental Non-Contact HR(V) Measuring System

**Figure 1:**
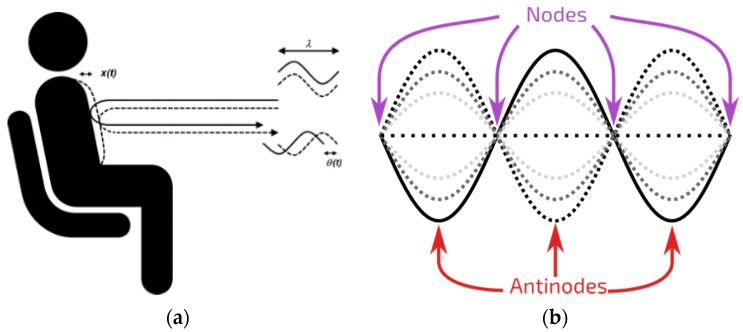
(**a**) Doppler effect: according to the Doppler effect, the phase change Δθ(t) of the reflected signal is proportional to motion of the measured location, scaled by wavelength of the signal Δθ(t)= 4πΔx(t)/λ, where Δx(t) is the chest displacement and λ is the wavelength of the transmitted signal; (**b**) Standing wave: the figure shows a possible sinusoidal standing wave. The different lines show the standing wave at different moments in time.

The experimental non-contact *HR(V)* measurement was carried out on the principle of the Doppler effect. While pumping blood through the cardiovascular system, the heart as well as veins undergo volumetric changes during every cardiac cycle. The information about cardiac cycle frequency is then reflected on the skin surface as a periodic movement of the subject’s chest/neck and is delayed compared to electrophysiological activity of the heart. Such displacement can be measured by means of sensors with adequate resolution, typically operating on the principle of Doppler effect (Figure 1a).

One such sensor uses the principles of radar. Since radar devices sense continuous electromagnetic (microwave radar operating at e.g., 2.4 GHz) or sound waves (ultrasonic sensor operating at e.g., 40 kHz) that are a reflection of an active transmission, they are considered to be active remote sensing units. Their sensitivity is directly correlated to their output power, where higher output power results in higher sensitivity [28]. While both the microwave and the ultrasonic sensor operate on the same principle, the technology itself is quite different. Ultrasonic sensors operate by emitting a burst of high frequency sound waves (therefore they need a medium to propagate) in a rapid succession and travel at the speed of sound, whereas microwave sensors are based on electromagnetic waves at higher speed compared to ultrasonic waves. As such, each will react differently to certain material. Generally speaking, the microwave sensor is less affected by environmental variables (e.g., temperature, condensing humidity, dust, etc.), however they are prone to dielectric noise, which can reflect an unwanted electromagnetic wave. As demonstrated in our previous work, the microwave sensor has a better signal to noise ratio at short measuring distances, whereas the ultrasound sensor performed better at larger distances [7,21].

In our study, the measurement of the Doppler effect was carried out using two ultrasound (US) transducers of the same type (model 400PT160). The used transducers are compact, lightweight (2 × 2 × 2 cm, 5 g), have a high sensitivity and are relatively insensitive to temperature and humidity variations. Other characteristics of the used transducers can be found on the manufacturers homepage [29].

The transducers were operated at two frequencies simultaneously, 40 kHz and 39 kHz, in order to reduce the occurrence of a standing wave, where adjacent points (peaks and nodes) are in phase with each other, so as to avoid the reflection of the wave from the skin in the node while the target is moving [30] (Figure 1b). In such an event information about the displacement due to cardiac activity would be lost.

The Sound Pressure Level (*SPL*) of the ultrasound radar used in the experiment was limited to *SPL* = 94 dB, with transducer terminal voltage 0.14 Vrms operating at both frequencies (*f*_1_ = 40 kHz, *f*_2_ = 39 kHz) simultaneously with transducers 25 cm from the body. As such, the SPL was within maximum allowed limits, which is set by International Commission on Non-Ionizing Radiation Protection guideline—ICNIRP to *SPL* = 100 dB at *f* = 40 kHz. This limit applies to continuous exposure to the general public for up to 24 h per day [31,32,33].

### 2.3. Sampling Device

The experimental non-contact measuring device was continuously sampled by means of a high-performance 24-bit/192 kHz A/D and D/A converter (A/D: 113 dB SNR, D/A: 117 dB SNR) E-MU 0404 system by Creative.

### 2.4. LabVIEW Application

**Figure 2:**
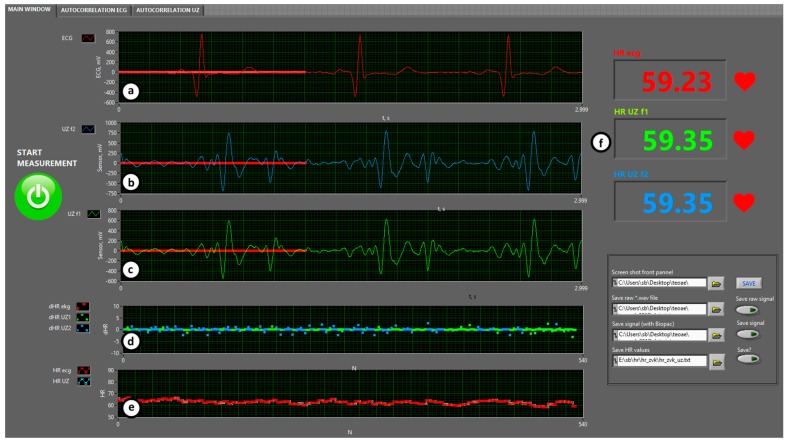
Front panel of the dedicated LabVIEW user interface: (**a**) reference III-Lead ECG signal; (**b**) processed ultrasound signal at *f*_1_ = 40 kHz; (**c**) processed ultrasound signal at *f*_2_ = 39 kHz. Note that US signals are delayed compared to ECG signal for about 200 ms because of the type and place of measurement; (**d**) measuring error, i.e., difference between the reference signal and experimental method signal instantaneous *HR* displayed on a diagram; (**e**) diagram of instantaneous *HR* of the two methods in question and (**f**) numerical display of instantaneous *HR* of all three signals.

For the purpose of data recording, data analysis and data representation in real time, the LabVIEW environment was selected. Figure 2 shows the front panel of the LabVIEW application. It is designed in a way to offer a simple overview of the most significant information, such as a comparison between the ultrasound signal at two frequencies and the reference signal, and the difference between the instantaneous *HR* values and the *HR* values throughout recording, both in the form of a graph on the one hand and a numerical representation on the other hand. All of this information is available to the observer in real time. Furthermore, the application enables one to record the acquired signal, providing the option of post-processing for later adaptation and improvements of the algorithm for new pathologies.

## 3. Experimental Setup

The experiment was carried out in three parts. Within the first part, a membrane of a loudspeaker was used, simulating the movement of a human body due to heart beat. The purpose of this part of the experiment was to determine the limits and characteristics of the non-contact measuring system in optimal conditions. The second part of the experiment was carried out on volunteers in laboratory conditions in two steps, before and after physical activity. The last part was conducted in a clinical environment on patients with different pathological conditions and under surveillance of medical staff.

Two transducers were used, one for transmitting and the other one for receiving the signal. Both sensors were directly connected to A/D device input/output without any additional hardware. In all cases, the transmitting ultrasound sensor was continuously transmitting at two different frequencies, *f*_1_ = 40 kHz and *f*_2_ = 39 kHz, in order to minimize the possibility of losing information due to standing waves. The used electrical transducer amplitude was 0.14 Vrms at the distance of 25 cm from the neck, resulting in *SPL* = 94 dB. With the purpose of meeting the Nyquist criteria of a processing system rule, the chosen sample rate for the ultrasound sensor was 96 kHz. The signals at the selected frequencies were mixed to zero in an intermediate frequency, split into the inphase (I) and quadrature (Q) channels and down sampled to 1 kHz for further processing. As indicated in our earlier work, the recorded signal is composed of several physiological signals, the most prominent being the low frequency respiratory signal and the high frequency heart rate signal. In order to extract Doppler heart beat signal, a Quadrature FM demodulator was used and further a low pass filter, a high pass filter and a band pass filer were applied [21].

The reference signal from the Biopac system was originally sampled at 1 kHz. The signal from the acquisition measurement device was already pre-filtered and ready to be displayed on the screen. For the purpose of further *HR* analysis, a cross correlation was used on the demodulated and filtered signals.

### 3.1. Test Signal Experiment

**Figure 3:**
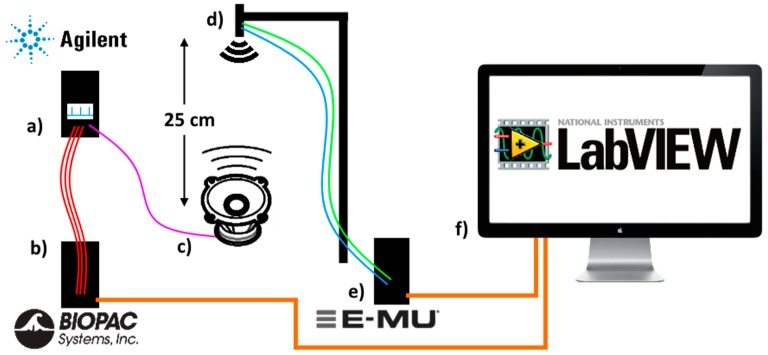
Test signal experiment consisting of (**a**) Agilent signal generator, generating a pulse signal with 0.5% duty cycle. The generated signal was connected to (**b**) Biopac ECG commercially available III-lead ECG system and (**c**) a standard speaker. The membrane of the speaker vibrated with the selected frequency (0.75 Hz to 3.6 Hz), mimicking a human torso. At the distance of 25 cm; (**d**) US transducers recorder the speaker signal via the (**e**) E-MU 24-bit/192 kHz A/D converter. Both acquired signals were analyzed by the (**f**) LabVIEW software.

The first milestone of the study was to establish a reliable non-contact measuring device, which will be able to repeatedly measure a stochastic physiological signal within known limits. The test signal (see Figure 3) was realized in the form of a square-wave pulse with a waveform generator 33522A by Agilent and a first order RC lowpass filter. The signal with a duty cycle set to 0.5% and amplitude 10 Vpp was simultaneously connected to an audio speaker and after attenuation of the signal also to electrodes of the commercially available III-lead ECG system on the other hand, with the purpose of comparison of both acquired signals in the LabVIEW application in real time. During the first part of the experiment a 2 min measurement was done measuring a pulse with a static frequency of 60, 120 and 180 beats per minute (1 Hz, 2 Hz, 3 Hz). During the second part of the experiment the frequency of the generated pulse signal was changed from 0.75 Hz to 3.6 Hz, which corresponds to 45 beats per minute and up to 216 beats per minute respectively. The signal connected to the audio speaker resulted in the vibrating of its membrane, which represented a very simplified model of a vibrating human torso.

### 3.2. Laboratory Experiment

The second part of the experiment was carried out in a controlled environment in a laboratory at the Faculty of Electrical Engineering, University of Ljubljana. The environmental conditions were controlled to be at the same level for all participants. The laboratory experiment was split in two parts. During the first part, the volunteers were laid on the bed and were asked to remain as relaxed as possible, to breathe in a calm manner and to minimize any movement in the neck area, e.g., speaking, swallowing, etc. The second part was carried out after 1 min of physical activity, during which the participants were doing squats, pushups or abs. Afterwards the volunteer was asked to return to bed and received the same instructions as in the first part. Measurements in both cases were conducted in a five minute interval. The two ultrasound transducers (one emitter and one receiver) were installed to a special holder at a distance of 25 cm from the subject’s neck. The beam of the ultrasound sensor was aimed to record the area around the external jugular vein. For the optimal sensing conditions, each subject was asked to tilt their head towards their left shoulder and expose the jugular vein. The ultrasound signal was continuously recorded by the E-MU 0404 device. The ECG electrodes were positioned to the subject’s left and right arm and the left leg. The signals from the electrode were continuously sampled by the Biopac MP150 acquisition system in combination with the ECG100C biopotential amplifier. The acquired signals were forwarded to a custom designed PC LabVIEW application.

### 3.3. Clinical Experiment

**Figure 4:**
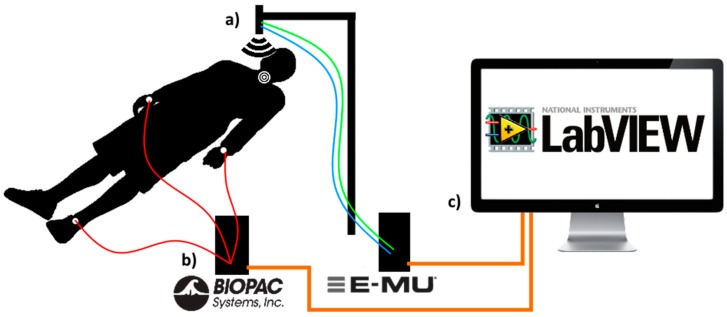
Experimental setup consisting of (**a**) ultrasound transducers measuring neck displacement due to volumetric changes of the external jugular veins during each cardiac cycle; (**b**) commercially available III-Lead ECG system (**c**) a dedicated LabVIEW application for signal recording and processing.

**Figure 5:**
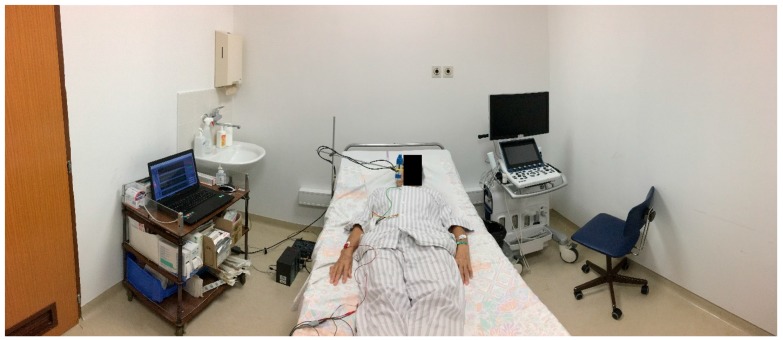
Clinical experiment non-contact measuring device setup.

The experiment was carried out in a controlled environment at the Clinical Department of Cardiology, University Medical Centre Ljubljana, under guidance of the medical staff. The environmental conditions were controlled to be at the same level for all participants. Other than that, the experiment was very similar to the first part of the laboratory experiment (without the physical activities), as presented in Figure 4 and Figure 5.

## 4. Results

For each part of the experiment, a statistical analysis was performed. For all recorded instantaneous *HR*, a difference between each ECG reference signal and each US experimental signals at *f*_1_ = 40 kHz and *f*_2_ = 39 kHz was calculated:

(1) $$\Delta HR_{i,f_{1}}=HR_{{\mathrm{ECG}}_{i}}-HR_{i,\mathrm{US}f_{1}}$$

(2) $$\Delta HR_{i,f_{2}}=HR_{{\mathrm{ECG}}_{i}}-HR_{i,\mathrm{US}f_{2}}$$

(3) $$\Delta HR_{i,f_{\mathrm{opt}}}=HR_{{\mathrm{ECG}}_{i}}-HR_{i,\mathrm{US}f_{\mathrm{opt}}}$$

where *i* is the consecutive sample iteration within the total sample size *n*. Also, the optimal instantaneous *HR* difference between the ECG signal and the US at *f*_1_ or *f*_2_ was calculated, based on the US value that was closer to the reference signal. Afterwards, the mean and standard deviation of the differences between adjacent signals were calculated at each frequency:

(4) $$\bar{\Delta HR_{f}}=\frac{\sum_{i=1}^{n}\Delta HR_{i,f}}{n}$$

(5) $$\sigma =\sqrt{\frac{1}{n-1}\overset{n}{\underset{i=1}{\sum }}{\left(\Delta HR_{i,\;f}-\bar{\Delta HR_{f}}\right)}^{2}}$$

Afterwards, a time domain *HRV* analysis was carried out to express the variance of the beat to beat interval sequence as an unordered set of intervals. This was based on the measured normal sinus to normal sinus (*NN*) interbeat intervals. The following commonly used time domain measures were calculated: the average of all NN intervals (*AVNN*), standard deviation of all *NN* intervals (*SDNN*), square root of the mean of the squares of the differences between adjacent *NN* intervals (*rMSSD*), and percentage of differences between adjacent NN intervals that are >20 msec and >50 msec (*pNN20* and *pNN50*). Additionally, a relative error between the reference signal and the optimal experiment signal is given.

(6) $$AVNN=\frac{\sum_{i=1}^{n}NN_{i}}{n}$$

(7) $$SDNN=\sqrt{\frac{1}{n-1}\overset{n}{\underset{i=1}{\sum }}{\left(NN_{i}-AVNN\right)}^{2}}$$

(8) $$rMSSD=\sqrt{\frac{1}{n-2}\overset{n}{\underset{i=2}{\sum }}{\left(NN_{i}-NN_{i-1}\right)}^{2}}$$

### 4.1. Test Signal

**Figure 6:**
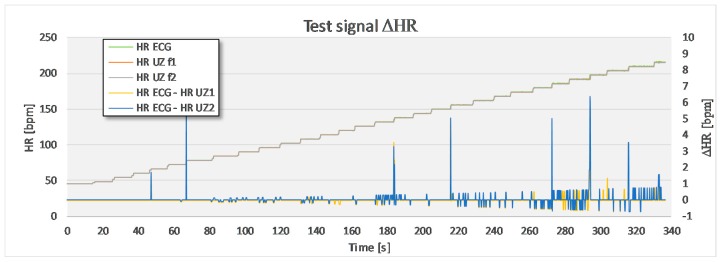
A comparison between the test signal, which is increased from 0.75 Hz (45 bpm) up to 3.6 Hz (216 bpm) in a random time interval, and the US signal. The largest deviations appear during the frequency change. Apart from that, the differences are kept below 1 bpm.

In the first step, a static signal at *f* = 1/2/3 Hz was recorded for 2 min. In the second step, the frequency of the signal produced by the generator was changed in steps of 0.1 Hz in a random manner, not taking any special care to the period between adjacent steps. A step of 0.1 Hz corresponded to, approximately, a change of 3 bpm. The calculated mean and standard deviations between mean instantaneous *HR* signal from the US and ECG measuring device are given in Table 1. In the case of static signal recording, there are only slight deviations between the reference signal and the experimental signal at the frequency 3 Hz (180 bpm). Larger deviations appear in the measurement where the frequency of the generated signal was changed dynamically. The mean and standard deviation of the differences between adjacent intervals between the US signal and the ECG signal are kept well below 1 bpm. The largest deviations within the measured signal, as visible from the Figure 6, occur exactly at the time of signal’s frequency change. These deviations occur as distinctive signal peaks and are of very short nature (≤0.2 s), therefore it is possible to filter them out.

### 4.2. Laboratory Experiment

A group of five male volunteers, aged between 25 and 33 years, participated within the laboratory part of the experiment. All participants but one did not have any known heart conditions. One of the volunteers has a known functional murmur. During the experiment we observed that the participants were breathing through their nose in a very shallow manner during the first part of the measurement. After the 1 min of physical activity some volunteers started to breath in a deep manner through their mouth, at least during the first half of the measurement. This also resulted in more frequent swallowing of saliva.

**Figure 7:**
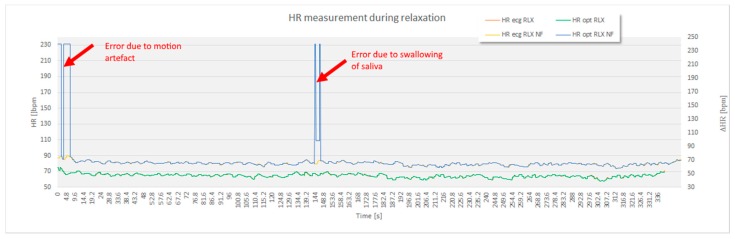
The non-filtered signal (NF) also included several disturbances caused by environmental factors. For the purpose of further analytical processing of the *HR* and time domain *HRV* markers (*AVNN*, *SDNN*, *rMSSD*, *pNN20* and *pNN50*), sections where the differences between the instantaneous ECG *HR* and US *HR* signal above 15 beats per minute were filtered out. The image demonstrates error in the US signal at the beginning of the recording while the subject was still moving, and error due to swallowing of saliva in the middle of the recording.

For the purpose of statistical analysis, all adjacent interval differences between the optimal experimental and the reference sensor method that were over 15 bpm were deleted from the recording (Figure 7) and excluded from statistical analysis. These parts of signal sections were though to present a non-physiologic part of the signal, such as unwanted body movement, swallowing of the saliva and surrounding noise. Apart from this, no signal modification took place.

**Figure 8:**
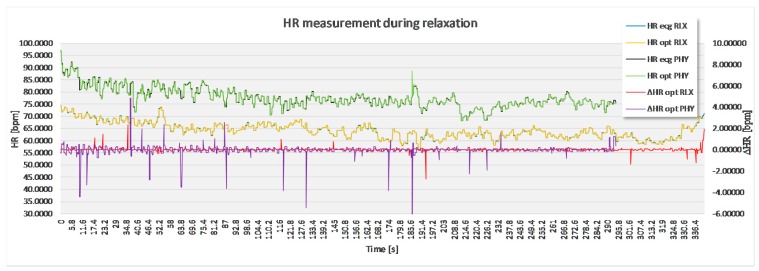
Recorded signal in laboratory experiment for subject 1.

**Figure 9:**
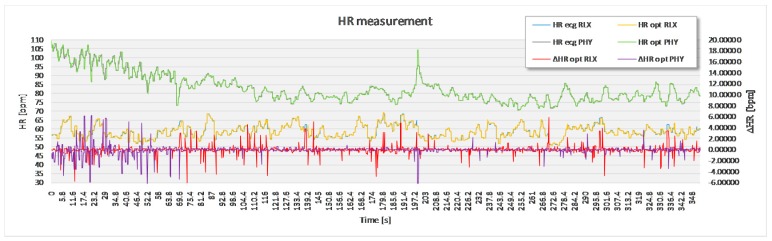
Recorded signal in laboratory experiment for subject 2.

**Figure 10:**
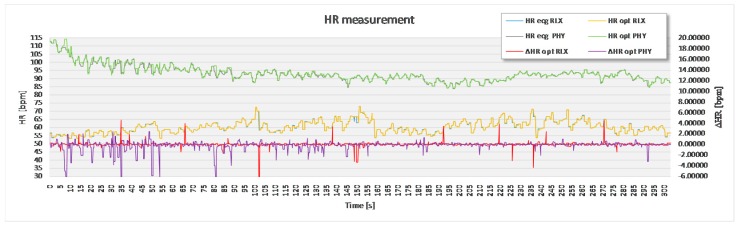
Recorded signal in laboratory experiment for subject 3.

**Figure 11:**
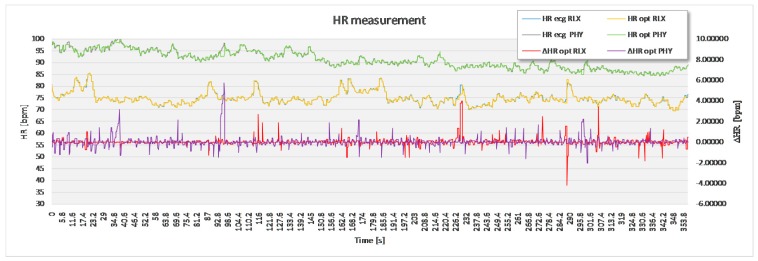
Recorded signal in laboratory experiment for subject 4.

**Figure 12:**
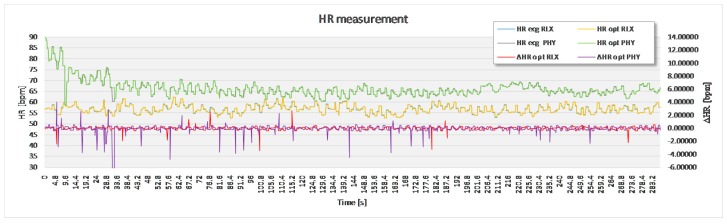
Recorded signal in laboratory experiment for subject 5.

The analysis based on the filtered signals of laboratory measurements for the five volunteers are provided in Table 2, Table 3, Table 4, Table 5 and Table 6. Also, the complete course of the recording for each subject is drawn in Figure 8, Figure 9, Figure 10, Figure 11 and Figure 12 (i.e., reference signal compared to experimental signal along with the difference between them for each interval). Each figure represents the signals measured during the relaxation period as well as the signals recorded after 1 min of physical activity. Note that the Figure 8, Figure 9, Figure 10, Figure 11 and Figure 12 only show the optimal signal compared to the reference one. In contrast, statistical analysis in Table 2, Table 3, Table 4, Table 5 and Table 6 include all instantaneous values, therefore the standard deviation is relatively high.

The *HR* and *HRV* statistical analysis is divided into two parts. The first part is based on data recorded during the relaxation phase and the second one to post physical activity. The *HR* part gives an insight to data for each frequency the transducers operated at (i.e., *f*_1_ = 40 kHz, *f*_2_ = 39 kHz), as well as for the optimal signal selection in real time at either frequency for each detected inter-beat interval when compared to the reference ECG measuring method, and also without comparison to the reference ECG method. In the latter case the optimal signal was calculated based on a moving average (last 20 instantaneous values of *HR*) of the ultrasound signal. In the last column, the percentage of the ejected signal due to signal noise is given. The signal noise was the result of body movement or saliva swallowing during the measurement. The *HRV* part presented in Table 3, Table 4, Table 5 and Table 6 on the other hand is only based on the optimal US signal.

Table 7 presents the average and the standard deviation of the instantaneous differences between the ECG and the US *HR* at *f*_1_ = 40 kHz, *f*_2_ = 39 kHz and the optimum frequency measurement for each inter-beat interval. As seen from the table, the algorithm’s ability to choose the measurement at the optimum frequency drastically improves the accuracy of the experimental method recording. Larger deviation appears at the measurement conducted after 1 min of physical activity, which is to be expected since the fluctuations in the signal are larger on the one hand, and subject’s had a more difficult time to remain completely still throughout the recording time.

**Figure 13:**
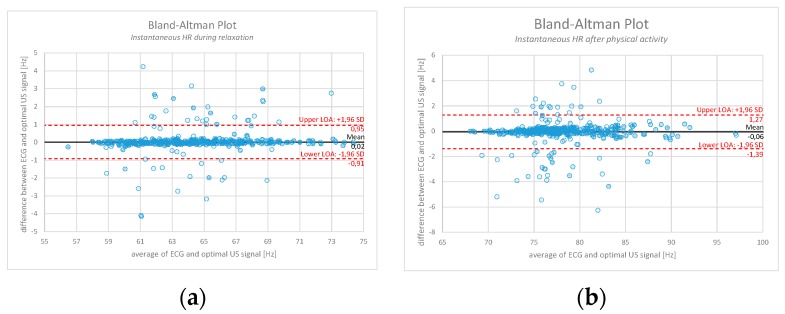
Bland-Altman plot comparison between the calculated instantaneous *HR* obtained from the ground truth ECG and the optimal US signal (where known unwanted artefacts have already been filtered out). (**a**) represents an example measurement during relaxation period (95.6% of the signal lies within the LOA); (**b**) represents an example measurement after physical activity (94.9% of the signal lies within the LOA).

With the purpose of validating the experimental method (after filtering out the known disturbances) against the reference one, Figure 13 represents a Bland-Altman plot example for a measurement conducted on an individual during relaxation (Figure 13a) and after 1 min of physical activity (Figure 13b). The comparison is done based on the calculation of instantaneous *HR* between the optimal US signal obtained with the moving average (therefore no ECG as a reference point) and the ground truth ECG signal. Over 1500 values of instantaneous *HR* are calculated, depending on percentage of ejected signal. In the first case, 95.6% of the signal is within the level of acceptance (LOA), whereas in the second case 94.9% of the signal is within the LOA.

### 4.3. Clinical Experiment

**Figure 14:**
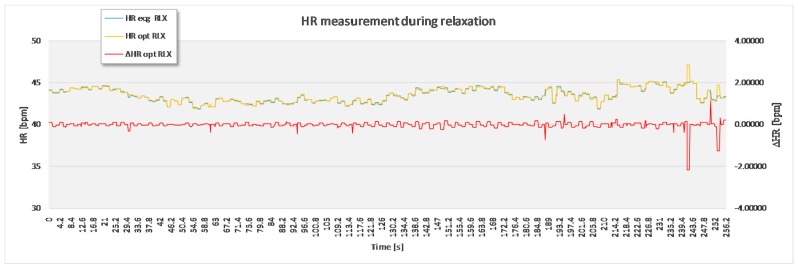
Sample of recorded signal on one of the patients in clinical environment.

Within the clinical part of the experiment, we conducted the measurement on a group of individuals, male and female, aged between 61 and 82 years. Each of the patients was diagnosed with a specific cardiac pathology. As measurements were done in real-life conditions, also more distractions were noticed, mainly noise created by people on the hall outside the experiment room talking, and from passing of other patients on foot, wheelchairs or being transported inside their hospital beds. Table 8 and Table 9 and Figure 14 demonstrate an example of results performed on a volunteer. This specific patient was diagnosed with atrial fibrillation with bradycardia ventricular response.

## 5. Discussion

The commonly clinically used measurement devices of heart physiological parameters (ECG and PPG) are reliable, but have specific limitations, which can be inappropriate for specific group of patients and can result in signal attenuation with time or even cause patient discomfort.

The results obtained within the experiment prove the possibility of non-contact measurement of heart physiological parameters on healthy subjects in a controlled laboratory environment with low electromagnetic noise and no sudden environment changes (temperature, humidity, noise, etc.). Even though the method required subjects to remain as still as possible throughout the duration of the measurement, the results also show that it is possible to perform measurements after physical activity, causing subjects to breathe through their mouth and swallow saliva more often than usual. Based on the measured signals it is possible to distinguish between disturbances and the physiological signal and furthermore filter out the noise. For the purpose of this paper, such noise was deliberately deleted from the both, experimental and reference recording in order to compare them in most objective manner. The percentage of deleted signal is provided in Table 2 and was between 2% and 17%, with the average value of the discarded signal at 6.8%. The actual percentage of the disposed signal was mostly caused by repeated swallowing of saliva and unwanted movement of the volunteer’s neck area. As minute movement signals resulting from vein contraction due to heart beat are measured, any such movement caused relatively large displacement of the sensor’s measuring position and, consequently, an unusable part of the signal. Instead of deleting the part of unusable signal, it could also be altered to correspond to the reference signal or the calculated moving average in points of maximum difference.

The results obtained from patients with a known cardiac condition indicate the possibility of conducting non-contact measurements in a clinical environment. Special attention however needs to be given to the correct calibration of the measurement device, stillness of the patients during the measurement and in ensuring that the jugular vein on the subject’s neck is fully exposed. Furthermore, work needs to be done on implementation of specific pathological characteristics of cardiac signals into the used PC algorithm in order to improve its accuracy.

Supported by the study results, the biggest advantage and novelty of the proposed non-contact measuring method compared to other studies is simultaneous operation of the US transducers at two different frequencies combined with the PC application algorithm’s ability to select the optimal signal in real-time. The comparison of the experimental US signal to the reference ECG signal presents the theoretically smallest deviation of the non-contact method with an existing algorithm. Since the end goal is measurement in a real environment, without the reference method against which the comparisons can be made, we have also shown the level of self-sufficiency of the non-contact experimental method by comparing instantaneous *HR* values to a moving average. As seen in the results, the signal deviation at a single frequency is relatively high. The mean value and standard deviation of instantaneous *HR* differences between the non-contact and reference method for all participants in the laboratory part of the experiment at *f*_1_ 40 kHz was *ΔHR* = 10.42 min^−1^ ± 28.49 min^−1^. On the other hand the values at the second frequency *f*_2_ = 39 kHz were *ΔHR* = 14.14 min^−1^ ± 34.39 min^−1^. After the best value between the two is selected, the values were *ΔHR* = 0.23 min^−1^ ± 0.61 min^−1^ for the case where the signal was compared to the reference one, and *ΔHR* = 0.31 min^−1^ ± 0.88 min^−1^ for the case with the calculated moving average. Such a decrease in error clearly shows the benefit of the proposed measuring system. In optimal conditions, this will result in scaling the differences between instantaneous ECG and US *HR* signal down to minimum, as presented in Table 7. Such an outcome will omit possible disturbances due to unwanted environmental noise. However, it will not address errors due to physical movement of the measured area. As the measuring ultrasound sensor’s position is fixed, any relative movement will result in measured signal deformation (in the presented case, up to 17% of the measurement). While it is safe to assume that patients can lay still for a short duration of a time, the same is not to be expected for longer periods of time, especially in an uncontrolled environment (e.g., during sleeping). Even though this remains common to non-contact methods overall, at this stage, this fact does represent a limitation to the proposed method in regards to use in a less controlled environment.

The highest deviations between the reference and the experimental signal at this point are the relative errors for the time domain *HRV* markers *rMSSD*, *pNN20* and *pNN50*. This is caused by the utilized algorithm in some cases causing the change in *HR* frequency of the US signal to appear in several steps, dividing it into smaller segments. Due to this, the affected *NN* intervals fall out of the 20 ms/50 ms domain respectively.

There is a lot of potential for the proposed non-contact *HR* and *HRV* measuring device, both in clinical (e.g., patients with burns, infants, patient with allergic reactions to ECG electrode gel, etc.) and non-clinical applications (e.g., elderly health care, etc.). Furthermore a lot of interest for such applications is also shown by other business segments, the biggest one being the automotive industry (e.g., keeping continuous track of the driver’s physiological condition). However, in order for these possibilities, the robustness of the proposed non-contact measuring methods need to be improved.

## 6. Conclusions

The purpose of this study was to perform a feasibility study of an assembled non-contact *HR* and *HRV* measuring device operating with US transducers. The feasibility study was performed in a laboratory setting on healthy volunteers and in a clinical environment on patients with known pathologies. The authors are fully aware that the entire study, at this stage, is primarily a proof of concept of non-contact *HRV*, more so than a fully accomplished research project. Nevertheless, it has been demonstrated that there is a considerable potential for further work based on the comprehensive research that has been already done.

In our future work, we will further improve the robustness of the proposed non-contact *HR* measuring device in terms of resistance to influence quantities. As such, we plan to reduce the impractical requirements for the subjects to remain still with their head tilted to one direction during the duration of the experiment. We also plan to improve the experimental method’s dependency on the reference signal. This will presumably be done by optimizing the averaging of the data points within a selected window size and excluding the points that lie outside a fixed fraction average (e.g., 20%). Additionally, most common cardiac pathologies will be further studied in order to improve the application algorithm accuracy for patients suffering from these pathologies, in cooperation with the medical staff of the Clinical department of cardiology, University Medical Centre of Ljubljana.

## Figures and Tables

**Table 1:** Mean and standard deviation of the differences between the instantaneous *HR* obtained with the experimental method at different frequencies (*f*_OPT_ = optimal frequency selected in real time) and the reference method.

Transducer Frequency	*f* = 1 Hz/2 Hz/3 Hz		*f* = 0.75–3.6 Hz	
	Mean *ΔHR* [bpm]	Std. Dev. *ΔHR* [bpm]	Mean *ΔHR* [bpm]	Std. Dev. *ΔHR* [bpm]
***f*_1_ = 40 kHz**	0/0/0.0080	0/0/0.0652	0.032	0.325
***f*_2_ = 39 kHz**	0/0/0.0083	0/0/0.0652	0.035	0.397
***f*_OPT_**	/	/	0.029	0.310

**Table 2:** *HR* statistical parameters for laboratory experiment, subject 1–5.

Transducer Frequency	During Relaxation					After Physical Activity				
	Average Value + Std. Dev. *ΔHR* (min^−1^)					Average Value + Std. Dev. *ΔHR* (min^−1^)				
	Sub. 1	Sub. 2	Sub. 3	Sub. 4	Sub. 5	Sub. 1	Sub. 2	Sub. 3	Sub. 4	Sub. 5
*f*_1_ = 40 kHz	0.11 ± 0.33	15.17 ± 35.77	3.33 ± 19.13	23.99 ± 53.95	9.50 ± 33.27	10.82 ± 36.52	12.86 ± 35.23	26.19 ± 54.68	26.44 ± 54.67	1.10 ± 9.11
*f*_2_ = 39 kHz	4.45 ± 25.61	41.46 ± 51.36	9.22 ± 36.07	12.94 ± 42.08	2.61 ± 16.85	26.46 ± 56.57	15.85 ± 40.54	2.26 ± 15.44	4.35 ± 23.78	6.03 ± 30.32
Opt. sig. with ECG	0.06 ±0.18	0.63 ± 1.98	0.13 ± 0.38	0.15 ± 0.33	0.17 ± 0.20	0.18 ± 0.44	0,38 ± 1.05	0.34 ± 72	0.25 ± 0.64	0.28 ± 0.48
Opt. sig. without ECG	0.16 ± 0.45	0.71 ± 2.10	0.16 ± 0.41	0.33 ± 1.20	0.18 ± 0.23	0.33 ± 0.16	0.56 ± 1.47	0.54 ± 0.92	0.38 ± 0.71	0.30 ± 0.50
Ejected signal (%)	2	17	5	4	11	3	9	7	7	3

**Table 3:** Representative time domain values of *HRV* measurements in a 5 min data set, during relaxation, subject 1–5, part 1.

Time Domain HRV Measures	During Relaxation									
	ECG					Optimal US Signal Compared to ECG Signal				
	Sub. 1	Sub. 2	Sub. 3	Sub. 4	Sub. 5	Sub. 1	Sub. 2	Sub. 3	Sub. 4	Sub. 5
*AVNN* (ms)	937.27	1029.28	1000.49	802.88	1059.13	937.27	1025.73	1000.50	803.07	1059.04
*SDNN* (ms)	46.95	62.61	61.28	27.41	35.34	46.82	68.11	61.30	27.33	35.34
*rMSSD* (ms)	24.29	48.50	46.81	15.03	38.06	23.64	66.38	45.55	15.40	37.86
*pNN20* (%)	41.77	69.71	68.15	13.71	67.66	39.04	62.96	66.67	14.14	69.97
*pNN50* (%)	2.13	32.94	23.97	1.02	21.66	2.09	29.63	24.05	1.00	21.92

**Table 4:** Representative time domain values of *HRV* measurements in a 5 min data set, during relaxation, subject 1–5, part 2.

Time Domain HRV Measures	During Relaxation									
	Optimal US Signal Not Compared to ECG Signal					Relative Error Compared/Not Compared to ECG (%)				
	Sub. 1	Sub. 2	Sub. 3	Sub. 4	Sub. 5	Sub. 1	Sub. 2	Sub. 3	Sub. 4	Sub. 5
*AVNN* (ms)	937.50	1026.86	1001.04	805.48	1059.36	0.00/0.02	0.34/0.23	0.00/0.06	0.02/0.32	0.01/0.02
*SDNN* (ms)	46.32	68.24	61.28	30.95	35.28	0.28/1.34	8.99/8.78	0.01/0.02	0.30/12.91	0.01/0.18
*rMSSD* (ms)	23.25	70.29	45.28	26.41	37.89	2.68/4.29	36.86/44.93	2.70/3.28	2.49/75.68	0.44/0.52
*pNN20* (%)	38.81	62.40	65.99	14.21	70.18	6.53/7.09	9.67/10.48	2.18/3.18	3.20/3.71	3.42/3.73
*pNN50* (%)	3.30	29.24	24.15	1.99	21.39	2.09/54.78	10.05/11.23	0.34/0.74	1.75/95.53	1.20/1.27

**Table 5:** Representative time domain values of *HRV* measurements in a 5 min data set, after 1 min physical activity, subject 1–5, part 1.

Time Domain HRV Measures	After Physical Activity									
	ECG					Optimal US Signal Compared to ECG Signal				
	Sub. 1	Sub. 2	Sub. 3	Sub. 4	Sub. 5	Sub. 1	Sub. 2	Sub. 3	Sub. 4	Sub. 5
*AVNN* (ms)	775.58	754.16	655.25	661.48	902.30	775.38	753.23	654.43	661.52	901.56
*SDNN* (ms)	39.79	62.24	32.40	25.96	41.30	39.66	62.99	32.94	26.11	41.56
*rMSSD* (ms)	22.74	20.83	12.03	8.02	30.42	22.53	21.32	13.15	11.57	30.55
*pNN20* (%)	37.43	32.12	8.16	0.42	48.51	37.99	30.08	10.24	0.77	44.90
*pNN50* (%)	1.71	3.01	0.19	0.83	8.01	1.68	2.92	0.20	0.84	7.94

**Table 6:** Representative time domain values of *HRV* measurements in a 5 min data set, during relaxation, subject 1–5, part 2.

Time Domain HRV Measures	After Physical Activity									
	Optimal US Signal Not Compared to ECG Signal					Relative Error Compared/Not Compared to ECG (%)				
	Sub. 1	Sub. 2	Sub. 3	Sub. 4	Sub. 5	Sub. 1	Sub. 2	Sub. 3	Sub. 4	Sub. 5
*AVNN* (ms)	776.64	755.04	656.10	662.55	901.34	0.03/0.12	0.12/0.12	0.12/0.13	0.00/0.16	0.08/0.11
*SDNN* (ms)	40.55	63.85	33.06	26.14	41.64	0.34/1.92	1.20/2.58	1.67/2.04	0.57/0.70	0.64/0.83
*rMSSD* (ms)	25.47	26.42	13.70	11.96	31.53	0.96/11.98	2.37/26.83	9.27/13.83	44.15/49.05	0.43/3.66
*pNN20* (%)	38.53	29.54	11.50	0.80	44.70	1.50/2.93	6.36/8.04	25.52/41.00	84.62/92.00	7.45/7.87
*pNN50* (%)	3.12	4.03	0.41	0.90	7.90	2.23/81.78	2.77/34.08	1.38/111.50	0.42/8.35	0.91/1.35

**Table 7:** Average and standard deviation of *HR* parameters for the laboratory group.

Transducer Frequency	During Relaxation	After Physical Activity
	Average Value + Standard Deviation *ΔHR* (min^−1^)	Average Value + Standard Deviation *ΔHR* (min^−1^)
*f*_1_ = 40 kHz	10.42 ± 28.49	15.48 ± 38.04
*f*_2_ = 39 kHz	14.14 ± 34.39	10.99 ± 33.33
Opt. sig. with ECG	0.23 ± 0.61	0.29 ± 0.67
Opt. sig. without ECG	0.31 ± 0.88	0.42 ± 0.75

**Table 8:** *HR* statistical parameters for clinical experiment.

Transducer Frequency	During Relaxation
	Average Value + Standard Deviation *ΔHR* (min^−1^)
*f*_1_ = 40 kHz	53.24 ± 84.66
*f*_2_ = 39 kHz	13.24 ± 48.60
Opt. sig. with ECG	0.41 ± 1.97
Opt. sig. without ECG	0.50 ± 2.00

**Table 9:** Representative time domain values of *HRV* measurements in a 5 min data set.

Time Domain HRV Measures	During Relaxation			
	ECG	Opt. Sig. with ECG	Opt. Sig. without ECG	Relative Error Compared/Not Compared to ECG (%)
*AVNN* (ms)	1373.51	1373.51	1369.31	0.05/0.31
*SDNN* (ms)	68.02	90.34	92.08	32.82/35.38
*rMSSD* (ms)	247.94	258.00	258.95	4.04/4.44
*pNN20* (%)	24.16	26.32	38.14	8.94/57.90
*pNN50* (%)	15.17	14.21	15.17	6.32/35.93
